# Nanofluidic Refractive-Index Sensors Formed by Nanocavity Resonators in Metals without Plasmons

**DOI:** 10.3390/s110302939

**Published:** 2011-03-04

**Authors:** Shih-Pin Tsai, Yao-Feng Ma, Ming-Je Sung, Ding-Wei Huang

**Affiliations:** Institute of Photonics and Optoelectronics, National Taiwan University, Taipei 10617, Taiwan; E-Mails: superpinpin3@msn.com (S.-P.T.); f94941019@ntu.edu.tw (Y.-F.M.); d96941003@ntu.edu.tw (M.-J.S.)

**Keywords:** resonators, sensors, refractive index

## Abstract

Nanocavity resonators in metals acting as nanofluidic refractive-index sensors were analyzed theoretically. With the illumination of transverse electric polarized light, the proposed refractive index sensor structure acts as a pure electromagnetic resonator without the excitation of surface plasmons. The reflected signal from the nanocavity resonators can be very sensitive to the refractive index of the fluids inside the nanocavities due to the enhancement of the electric field of the resonant mode inside the cavities. Such a sensor configuration can be a useful tool for probing the refractive index change of the fluid inside the nanocavities using the spectral, angular or intensity interrogation schemes. The wavelength sensitivity of 430 nm/RIU, angular sensitivity of 200–1,000 deg/RIU and intensity sensitivity of 25.5 RIU^−1^ can be achieved in the proposed sensor configuration.

## 1. Introduction

Although nanostructured metals which utilize the excitation of surface plasmons have been widely used as facilitating tools for probing surface interactions [1,2] and enhancing the nonlinear effects near metal surfaces [3], the enhancement of the electric fields in some nanostructured metals without plasmons have been reported for enhancing the transmission of light [4], reducing the reflection of light [5], or enhancing the absorption of light in metal nanostructures [6]. In this context, the use of nano-cavity resonators in metals as refractive index sensors was proposed and studied. With the illumination of transverse electric (TE) polarized light, the proposed structures act as pure electromagnetic cavity resonators without the excitation of surface plasmons. It is well-known that the electric field in a cavity resonator can be significantly enhanced inside the resonator and the corresponding field enhancement is sensitive to several factors, such as the geometry of the resonator, the coupling of light into/out of the resonator, the optical loss in the resonator, and the refractive index of the fluid filling the resonator. In our study, the feasibility of using the proposed nano-cavity resonators in metals as refractive index sensors and the performance of such a sensor configuration were evaluated.

## 2. Design and Experiments

**Figure 1.**
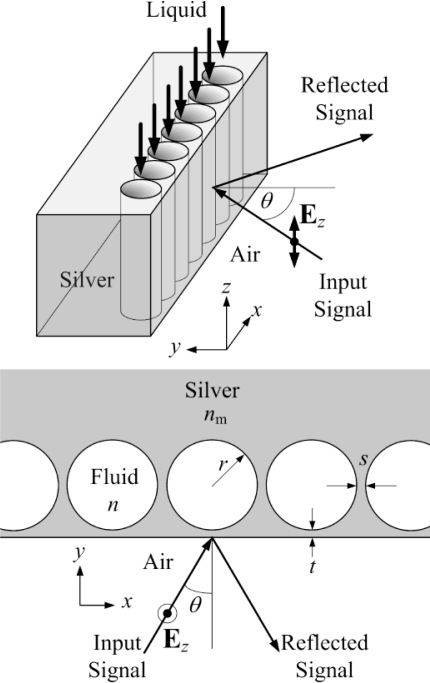
Schematic of the proposed sensor configuration based on the nanocavity resonators in metal.

Figure 1 shows the schematic of the proposed refractive index sensor configuration. The sensor was formed by closely spaced nanocavities embedded in the metal (silver). The spacing *s* between any two adjacent nanocavities was chosen to be 10 nm so as to maintain sufficient mechanical strength to support the thin metal film at the surface. Meanwhile, the spacing between the cavities and the metal surface was reserved for controlling the coupling of the illuminated light into the cavities. Note that the nanostructure is assumed to be invariant in the *z*-direction. The input optical signal was incident at an angle *θ* on the metal surface and the change in the reflected signal was then used to indicate the refractive index change of the fluid filling the cavities. This nanostructure can be fabricated by coating metal films over closely spaced latex nanocylinders, followed by removal of the latex nanocylinders with suitable organic solvents so as to form the required nanocavities. In order to estimate the required radius of the nano-cavities for the resonance of electromagnetic waves at the target wavelength, a simplified case of a single isolated cylindrical cavity embedded in a perfect conductor was assumed first. The analytic solution of the *m*^th^ order resonant mode in such a single isolated cylindrical cavity resonator is given by Expression (1):

(1) $$r_{\mathrm{ml}}=p_{\mathrm{ml}}\;\lambda /2\pi n$$

where *r_ml_* is the radius of the cylindrical cavity required for the *m*^th^ order resonant mode, *λ* is the target wavelength, *n* is the refractive index of the fluid filled inside the cylindrical cavity, and *p_ml_* represents the *l*^th^ zero of the *m*^th^ order Bessel function of the first kind (e.g., *p*_10_ = 2.4048, *p*_11_ = 3.8317, *etc.*) [7,8]. Based on this simplified model, the estimated radius of the nanocylinder cavities filled with pure water (*n* = 1.33) for the fundamental resonant mode is $r_{01}=p_{01}\frac{\lambda }{2\pi n}\approx 182\;\text{nm}$ at the wavelength of 632.8 nm. As the spacing between the nanocylinder cavities becomes smaller, the cavities may form coupled cavities and the coupling of the electromagnetic fields between the nanocylinder cavities may result in a red shift of the resonant wavelength due to the larger effective dimension of the resonant cavity contributed by the adjacent nanocylinder cavities. If the nanocylinder cavities are closely packed (*s* = 0) or even merged (*s* < 0), the coupling between adjacent nanocylinder cavities becomes even stronger. For an extreme case when adjacent nanocylinder cavities are completely merged with each other and the boundary between them vanishes, the structure may evolve into a Fabry-Pérot cavity with a cavity length equal to the diameter of the nanocylinder. In this case, the relation between the resonant wavelength of the fundamental resonant mode and the radius of the nanocylinder *r*_FP_ would become:

(2) $$r_{\text{FP}}=\lambda /4n$$

For the resonant wavelength at 632.8 nm, the required radius of the nanocylinder is *r*_FP_ = *λ*/4*n* ≈ 119 nm (for *n* = 1.33) for such an extreme case. In our study, the spacing (*s*) between adjacent nanocavities is kept for the purpose of not only providing sufficient mechanical strength but also allowing the electromagnetic fields in the nanocavities to be coupled with each other. Therefore, the actual radius of the nanocylinder cavity may be between the two extreme cases, *i.e*., within the range *r*_FP_ < *r* < *r*_10_, for the target resonant wavelength. In our simulation, the physical radius of 140 nm was determined by considering the spacing *s* = 10 nm. In order to allow the light to be efficiently coupled into the resonant cavities, the spacing (*t*) between the cavities and the metal surface should be carefully chosen. Note that a smaller spacing (*t*) implies a stronger coupling effect. As the resonant fields in the cavity gain the optical power from the incident light through the coupling, it may suffer not only the power loss in the cavity due to the light absorption by the metal but also the power loss of being coupled out through the metal at the same time. The refractive index of silver is *n*_m_ = 0.135 + 3.98*i* at 632.8 nm [9] and the corresponding penetration depth is *d*_p_ = *λ*/[2π · Im(*n_m_*)] = 632.8/(2π · 3.98) ≈ 26 nm. Accordingly, the optimal value of the spacing is found to be *t* ≈ 8.5 nm which is approximately 1/3 of the penetration depth. After the dimensions of the refractive index sensor configuration were decided, the reflection spectra for the normal incident light can be obtained by using the rigorous coupled-wave analysis (RCWA) technique [10].

**Figure 2.**
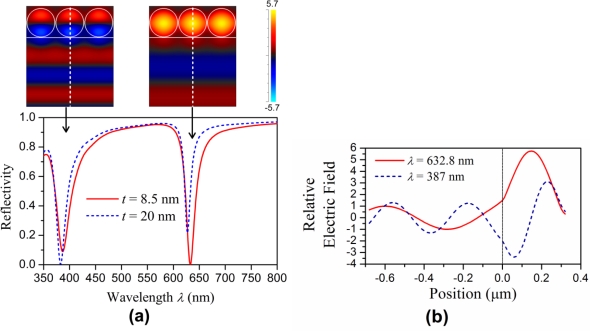
**(a)** Reflection spectrum for the normal incident light. The two insets show the relative electric field distributions for the two spectral dips, respectively. **(b)** Relative electric field distributions along the *y* axis (the dashed cross-section lines shown in the insets) for the two spectral dips with *t* = 8.5 nm.

In the RCWA calculation, a unit cell (*i.e*., one period) of the periodic structure, which is 290 (= 2 × 140 + 10) nm by 1 μm, is discretized into 2,900 × 10,000 grid points with a step size of 0.1 nm in both the *x* and *y* axes so as to allow the staircase approximation of the circular boundary of the nano-cylinder to be as smooth as possible for minimizing the numerical error. Meanwhile, a harmonic order up to 35 is used in the series expansion formula for the periodic structure in the *x* axis so that a sufficiently accurate result can be obtained while not exceeding the computer memory limit and maintaining a reasonable calculation time. After solving the eigenvalue of the RCWA formula in matrix form, the relative electric filed distribution and the reflectivity *R* over the spectral range 350–800 nm were obtained. Figure 2(a) shows the calculated reflection spectra of the normal incident light for the two cases *t* = 8.5 nm (solid line) and *t* = 20 nm (dashed line). Note that the refractive index of the fluid inside the nanocavities was fixed at 1.33 in this case. In Figure 2(a), there are two dips in the reflection spectrum: one at 632.8 nm and the other at 387 nm, which correspond to the fundamental resonant mode and the first order resonant mode in the nanocavities, respectively. The relative electric field distributions corresponding to these two spectral dips for *t* = 8.5 nm are shown in the insets of Figure 2(a).

Figure 2(b) further shows the relative electric field distributions along the *y* axis for the resonant modes at 632.8 nm (solid line) and 387 nm (dashed line) with the corresponding local electric field enhancement factors of 5.7 and 3.1, respectively. Because the spacing *t* = 8.5 nm (solid line in Figure 2) is not an optimal value for the first order mode, the corresponding spectral dip at 387 nm for the first order mode is shallower and wider than that at 632.8 nm for the fundamental mode. On the other hand, for the spacing *t* = 20 nm (dashed line in Figure 2), the optimal coupling strength for the first order mode to sustain the strongest resonance can be achieved, thus a deeper dip at 387 nm can be obtained. Based on these observations, in order to obtain a good discrimination capability of the refractive index sensor, the deeper and narrower dip at 632.8 nm for the fundamental resonant mode with *t* = 8.5 nm was used. Further numerical simulations were then conducted to understand the behaviors of the resonant fields and the corresponding reflected signals under different wavelengths and incident angles of the input signal as the refractive index of the fluid was varied.

## 3. Result and Discussion

**Figure 3.**
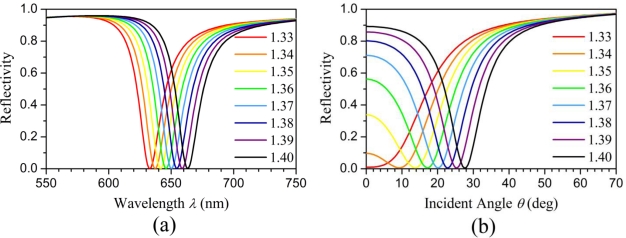
**(a)** The reflection spectrum for the normal incident light, and **(b)** the reflectivity for the oblique incident light at 632.8 nm as functions of the refractive index of the fluid.

Figure 3(a) shows the calculated reflection spectra as functions of the wavelength of the normal incident input signal for different refractive indices of the fluid inside the cavity ranging from 1.33 to 1.4. A clear red shift in the reflection spectra can be observed. The resonant wavelength has an approximately linearly proportional relation with the refractive index of the fluid. When the wavelength of the input optical signal is fixed, the behavior of the fundamental resonant mode in the nano-cavities may also change when the incident angle is varied.

**Figure 4.**
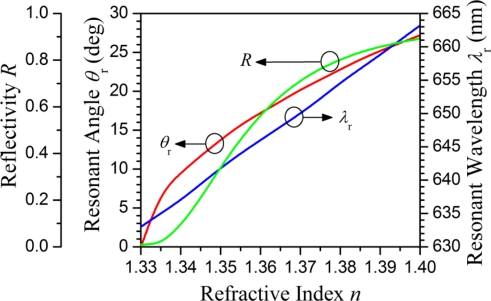
The resonant wavelength (*λ_r_*) for the normal incident light, the resonant angle (*θ*_r_) for the oblique incident light at 632.8 nm, and the reflectivity (*R*) for the normal incident light at 632.8 nm as functions of the refractive index of the fluid.

Figure 3(b) shows the calculated reflectivity as functions of the incident angle of the input signal at 632.8 nm for different refractive indices of the fluid inside the cavities. The resonant angle increases with the refractive index of the fluid. Meanwhile, the intersection points of the curves with the *y*-axis shown in Figure 3(b) represent the reflectivity for the normal incident light at 632.8 nm for different fluid refractive indices. The reflectivity *R* increases with the refractive index of the fluid inside the cavities. Figure 4 shows three curves which correspond to the resonant wavelength (*λ*_r_) for the normal incident light, the resonant angle (*θ*_r_) for the oblique incident light at 632.8 nm, and the reflectivity (*R*) for the normal incident light at 632.8 nm, respectively as functions of the refractive index of the fluid inside the cavities. The wavelength sensitivity *S_λ_* = ∂*λ_r_*/∂*n* is approximately 430 nm/RIU. Unlike the resonant wavelength, the resonant angle *θ*_r_ does not have a linearly proportional relation with the refractive index of the fluid inside the cavities. The slope of the resonant angle curve is steeper for the refractive index of the fluid within the range of 1.33–1.34 and become nearly constant for the refractive index of the fluid higher than 1.34. Accordingly, the corresponding angular sensitivity *S_λ_* = ∂*λ_r_*/∂*n* is ∼1,000 deg/RIU for n = 1.33–1.34 and ∼200 deg/RIU for n = 1.34–1.4. Like the curve of the resonant angle, the reflectivity curve is not a straight line, but rather a nonlinear curve. The intensity sensitivity *S_I_* = ∂*R*/∂*n* is approximately 25.5 RIU^−1^ at the point of the reflectivity curve with the steepest slope.

Compared to the wavelength sensitivity of a typical SPR based sensor (*S_λ_* = 1,000–10,000 nm/RIU), the wavelength sensitivity of the nanocylinder cavity resonator proposed in this study (*S_λ_* ∼ 430 nm/RIU) is much smaller due to the fundamental difference between their resonance conditions: the resonance condition for a typical SPR based sensor is dominated by the strongly wavelength-dependent (highly dispersive) dielectric constant of the metal, while the resonance condition for the metal nanocavity resonator proposed in this study is dominated by the dimension of the nanocavity and the resonance order. Although the wavelength sensitivity is much smaller than typical surface plasmon resonance (SPR) based sensors, the angular sensitivity and intensity sensitivity of the proposed nano-cavity resonators can be comparable or even higher than those of conventional SPR-based sensors. According to the results of our study, such a refractive index sensor configuration based on nano-cavity resonators can be a useful tool for probing the refractive index change of the fluid using the spectral, angular or intensity interrogation schemes.

## 4. Conclusions

Nanofluidic refractive-index sensors formed with nanocavity resonators in metals were analyzed theoretically. Without the excitation of surface plasmons due to the illumination of transverse electric polarized light, the proposed refractive index sensor structure acts purely as a cavity resonator. The reflected signal from the nanocavity resonator is very sensitive to the refractive index of the fluid inside the nanocavities due to the resonance of the electromagnetic waves inside the cavities. Such a sensor configuration can be a useful tool for probing the refractive index change of the fluid inside the nano-cavities using either the spectral, angular or intensity interrogation schemes. For spectral interrogation near 632.8 nm, the wavelength sensitivity is approximately 430 nm/RIU. For angular interrogation at 632.8 nm, the angular sensitivity is 200–1,000 deg/RIU. For the intensity interrogation scheme at 632.8 nm, the intensity sensitivity is 25.5 RIU^−1^.
